# Non-Coding RNA in Systemic Sclerosis: A Valuable Tool for Translational and Personalized Medicine

**DOI:** 10.3390/genes12091296

**Published:** 2021-08-24

**Authors:** Marta Rusek, Dorota Krasowska

**Affiliations:** 1 Department of Dermatology, Venereology and Pediatric Dermatology, Laboratory for Immunology of Skin Diseases, Medical University of Lublin, 20-080 Lublin, Poland; dorota.krasowska@umlub.pl; 2 Department of Pathophysiology, Medical University of Lublin, 20-090 Lublin, Poland

**Keywords:** long non-coding RNAs, systemic sclerosis, autoimmunity

## Abstract

Epigenetic factors are heritable and ultimately play a role in modulating gene expression and, thus, in regulating cell functions. Non-coding RNAs have growing recognition as novel biomarkers and crucial regulators of pathological conditions in humans. Their characteristic feature is being transcribed in a tissue-specific pattern. Now, there is emerging evidence that lncRNAs have been identified to be involved in the differentiation of human skin, wound healing, fibrosis, inflammation, and immunological response. Systemic sclerosis (SSc) is a heterogeneous autoimmune disease characterized by fibrosis, vascular abnormalities, and immune system activation. The pathogenesis remains elusive, but clinical manifestations reveal autoimmunity with the presence of specific autoantibodies, activation of innate and adaptive immunity, vascular changes, and active deposition of extracellular matrix components leading to fibrosis. The use of multi-omics studies, including NGS, RNA-seq, or GWAS, has proposed that the non-coding genome may be a significant player in its pathogenesis. Moreover, it may unravel new therapeutic targets in the future. The aim of this review is to show the pathogenic role of long non-coding RNAs in systemic sclerosis. Investigation of these transcripts’ functions has the potential to elucidate the molecular pathology of SSc and provide new opportunities for drug-targeted therapy for this disorder.

## 1. Introduction

Systemic sclerosis (SSc) is an autoimmune complex connective tissue disease characterized by three main features: impaired angiogenesis and vasculopathy, immune system dysregulation, and progressive fibrosis of the skin and internal organs [1,2]. The pathological process is complicated and remains not fully elucidated; thus, the early diagnosis and efficient treatment is challenging in a clinical setting. Examination of extension of skin involvement, the intensity of its hardening, and clinical features reveals two forms of SSc: limited cutaneous SSc (lcSSc) and diffuse cutaneous SSc (dcSSc) [3]. LcSSc is rarely associated with internal organ fibrosis and dcSSc is more often connected to organ manifestation, such as intestinal lung disease (ILD), renal crisis, and myocardial fibrosis [3,4]. Because of vascular and fibrotic organ damage progression, morbidity and mortality are close to 25% in the first 5 years after SSc diagnosis [5,6].

In the past, the explanation of autoimmune system disease development was limited to the presence of specific proteins such as antinuclear antibodies (ANA), characteristic for autoimmune diseases such as scleroderma, lupus, mixed connective tissue disease, and autoimmune hepatitis [7]. However, thanks to high-throughput sequencing technologies, a growing number of evidence indicates the role of coding and non-coding RNAs in regulation at the transcription and post-transcription level and epigenetic modification in several human diseases, especially autoimmune and inflammatory diseases, as well as cancer [8]. Therefore, understanding the underlying molecular mechanisms is crucial to find the primary triggers of autoimmune disease development. The pathogenesis process of SSc involves abnormal regulation of the immune system [9], the activity of immune cells, and the fibrosis process [8]. The pathological mechanism starts with microvascular injury due to a critical imbalance between proangiogenic and antiangiogenic factors [10]. Therefore, endothelial cell injury promotes autoimmune reactivity and perivascular injury, inducing activation of fibroblasts, tissue fibrosis, and, therefore, scleroderma-like disease [11,12].

Systemic sclerosis is characterized by activated fibroblasts and excessive accumulation of collagen fibers, fibronectins, and other extracellular matrix components, resulting in organ dysfunction and tissue fibrosis [10,13]. The process starts with transforming growth factor-β (TGF-β) release and other cytokines. Increased levels of Th2 cytokines, such as TGF-β and IL-13, have been found in the tissues of SSc patients [12,13,14]. Therefore, they induce the differentiation of fibroblast and infiltration fibroblast in the activated form [15]. Fibroblast transition to myofibroblasts is suggested to be a significant step in SSc pathogenesis [1]. The population of myofibroblasts cells is heterogeneous that derives from several different cellular precursors [16]. They are activated by various signaling pathways such as TGF-β, Wnt/β-catenin, which triggers canonical Smad 2/3 and non-canonical Smad 4 signaling pathways, as well as a platelet-derived growth factor, and hedgehog signaling [12,16]. Moreover, B lymphocytes are activated by Th2-derived cytokines, such as IL-4 and IL-5, and IL-6, derived from macrophages to produce several types of autoantibodies that result in vascular endothelial cell damage, tissue ischemia, chronic inflammation, and eventually tissue fibrosis [12,17]. A lack of typical signs and symptoms of SSc clinically may cause difficulties in its diagnosis. Therefore, the delay in the diagnosis and treatment of SSc may lead to uncontrolled disease progression [18]. Thus, explaining the contribution factors, finding, and recognizing possible early biomarkers of skin and organ involvement to prevent further damage of SSc is crucial for a patient [18].

Over the past few years, numerous factors have been demonstrated to contribute to the pathogenesis of the disease, including genetic susceptibility, environmental factors such as viral infections [19,20], and epigenetic modifications in genes related to the pathogenesis of SSc [2], implicating that epigenetics may play a significant role in the disease development [2,21,22]. Several experimental studies revealed that specific gene sets are expressed in the peripheral blood and skin of SSc patients [23,24,25,26], which are associated with fibrosis-related pathways such as TGF-β and Wnt/β-catenin signaling pathways and collagen synthesis (COL4A3, COL4A4, COL5A2, COL13A1, COL22A1, CTGF) [27], immunologic response, B-cell signaling (BANK1), interleukin signaling (IL12A, IL12RB1, IRAK1), IFN signaling (IRF4, IRF5, STAT4), activated macrophages, chemokines, as well as keratin-related pathways (keratin genes) [12,16,23,27,28,29,30,31]. It is noteworthy that epigenetic features are factors regulating gene expression without modifying the underlying DNA sequence, described by DNA methylation and hydroxymethylation, histone modifications, and non-coding RNAs [32].

Long non-coding RNAs (lncRNAs) are functional transcripts longer than 200 nucleotides (nt) that do not translate into proteins [33] and are diverse, including antisense RNAs, long intergenic non-coding RNAs (lincRNAs), and pseudogenes [6]. An interactive database (NONCODE) that aims to present the complete collection of ncRNAs has compiled over 90,000 human lncRNA genes and over 140,000 human lncRNA transcripts [34]. In addition, Hon et al., using FANTOM5 cap analysis of gene expression (CAGE) data, have integrated multiple transcript collections to develop a comprehensive atlas of 27,919 human lncRNA genes [35]. Moreover, they built expression profiles across 1829 samples from the major human primary cell types and tissues [35].

LncRNAs are involved in regulatory functions in cell physiology (chromatin remodeling, gene transcription, and translation) and cellular signaling [33,36]. On the other hand, the dysregulated lncRNAs, as a result of environmental factors and genetic mutations, are associated with several pathologies such as neurological disorders (Alzheimer’s disease) [37], cardiovascular disease [38], metabolic syndrome (glucose, lipid, and bile acid homeostasis disruptions) [39], and cancer [36]. Therefore, lncRNAs have been implicated in the pathogenesis of autoimmune diseases such as psoriasis [40], systemic lupus erythematosus [41], rheumatoid arthritis [42], type 1 diabetes, and particularly in SSc [43]. The comprehensive analysis of lncRNAs expression in the context of SSc pathogenesis is still unexplained [44]. Moreover, compared to coding genes, lncRNAs demonstrate higher tissue specificity in their pattern of expression [44]. The specific role of lncRNAs still remains unknown. However, the studies demonstrate their essential role in regulating and shaping the genome [6,45,46]. Recent studies indicate that lncRNAs are associated with developing diverse immune cells and controlling the dynamic transcriptional processes [47]. Understanding lncRNAs regulation of genes extends our knowledge of various pathologies [8]. In addition, identifying informative biomarkers could potentially explain the underlying pathogenic mechanisms in autoimmune diseases, including SSc development.

In this review, we highlighted the biological role of lncRNAs and examined their function in the pathogenesis and development of immune-related diseases, particularly in systemic sclerosis. Additionally, we discussed their potential role as biomarkers and therapeutic agents.

## 2. Non-Coding RNAs

Non-coding RNAs (ncRNAs) are characterized by definition as transcripts that do not encode proteins [48] but are functional in translation as well as in splicing events [48,49]. However, recent studies using next-generation RNA sequencing (RNA-seq) have demonstrated that the number of lncRNAs is higher than the number of genes encode proteins. Moreover, they suggested a few lncRNAs with short open reading frames (ORFs) coded for polypeptides [50,51] that have biological activities [52]. However, the biological significance of these hidden polypeptides remains unexplained [53].

The group of ncRNAs encodes 98% of the human genome [34]. Thus, they are essential regulators of biological homeostasis by modulating the gene expression at transcriptional, post-transcriptional, and epigenetic levels [54,55]. Noteworthy, increasing research data shows that altered expression levels of ncRNAs play a significant role in the etiopathogenesis of neuronal disorders, immune responses, and cancer [47], and may be associated with poor clinical outcomes [52]. In addition, their tissue specificity and condition-specific expression patterns indicate that lncRNAs are potential biomarkers and provide a rationale to target them at the clinical setting [47], as well as possibly valuable targets for more effective treatment [52].

**Figure 1:**
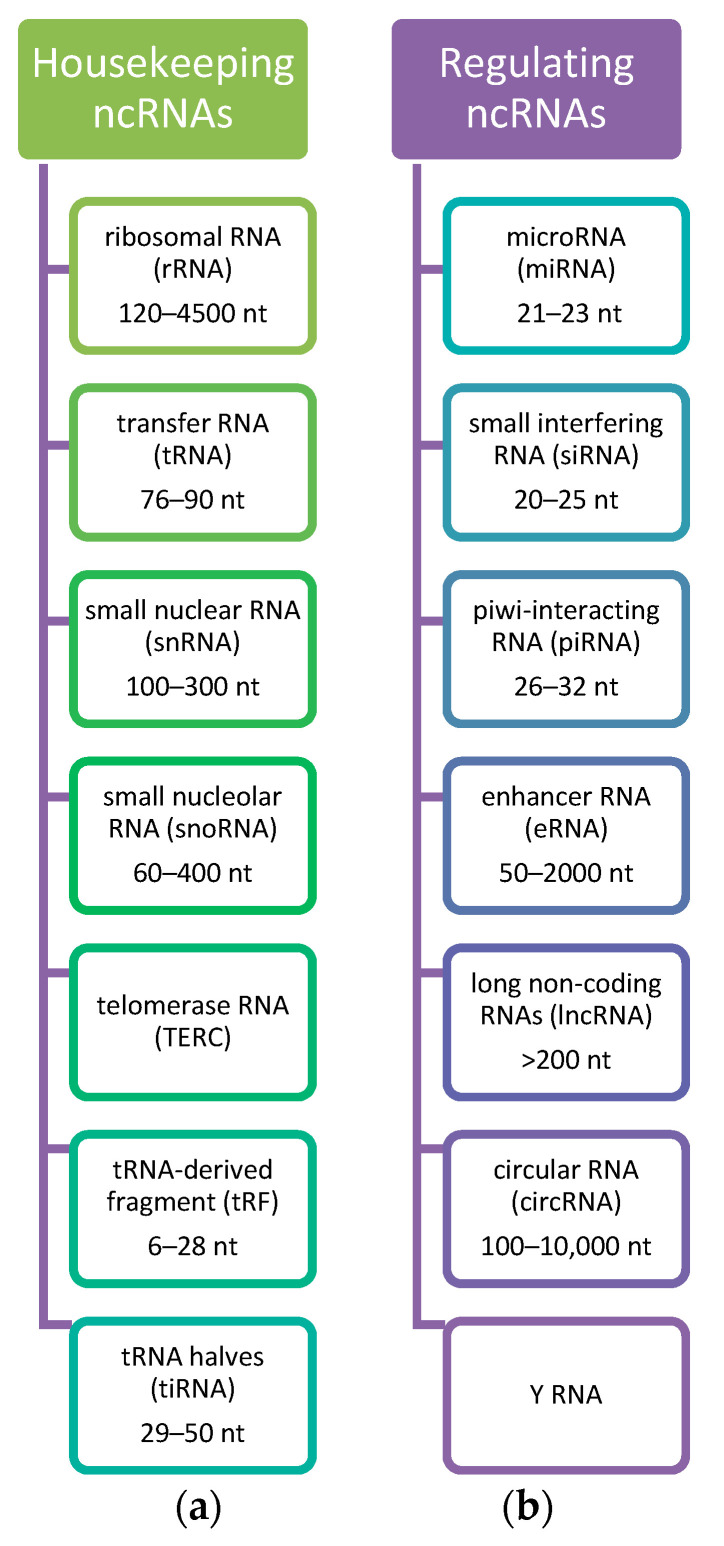
Classification of ncRNAs. Considering the role of ncRNAs, they are divided into (**a**) Housekeeping ncRNAs that are expressed in cells, mainly regulate cellular functions and (**b**) Regulatory ncRNAs considered as regulators of gene expression at transcriptional, post-transcriptional, and epigenetic levels [49,56,58]. Based on the [49].

These ncRNAs may be categorized and classified into different groups according to size and their function. Thanks to the advances in deep sequencing technologies, ncRNAs are classified into small ncRNAs (<200 nt, including piwi-associated RNAs, endogenous short-interfering RNAs, microRNAs, and Y-RNAs), and long ncRNAs (lncRNAs, >200 nt) [49]. By function, they are categorized into ribosomal RNAs (rRNAs), transfer RNAs (tRNAs), circular RNAs (circRNAs), small nuclear RNAs (snRNAs), small nucleolar RNAs (snoRNAs), microRNAs (miRNAs), long non-coding RNAs (lncRNAs), transcription initiation RNAs (tiRNAs), and piwi-interacting RNAs (piRNAs) [54,56,57]. The classification of ncRNAs is presented in Figure 1.

### 2.1. LncRNAs

The global network of ncRNAs includes several different types of molecules from multi-omics datasets presented in distant subcellular locations [49]. The broad term lncRNAs includes many different types of RNA, and displays a range of genomic structures and relationships to the coding transcriptome [59]. The human genome contains ~51,382 lncRNA genes [60]. Most of the lncRNAs are transcribed by RNA polymerase (Pol) Pol II/Pol I, and only a few are transcribed by RNA Pol III [61]. They may be polyadenylated and can be located within nuclear or cytosolic fractions [62]. Moreover, it has been observed that the secondary structure of lncRNAs is well-conserved, which plays a role in coordinating RNA–RNA, RNA–protein, and RNA–DNA interactions [63], and potentially regulate their targets [51]. In addition, lncRNAs are able to interact with miRNA, which may serve as sponge-like molecules to inhibit miRNAs-mediated functions [12,64].

The classification of lncRNAs is based on their position to nearby protein-coding genes; thus, it is intergenic, intronic, antisense, sense, enhancer, and bidirectional [9,12,65]. Intergenic lncRNAs are located between protein-coding genes with the separation of transcriptional units. Based on the genomic and epigenomic classification, the most intergenic lncRNAs originate from enhancers rather than promoters [35]. Moreover, they are tissue-specific compared with coding genes and co-expressed with their neighboring genes [66]. Enhancer lncRNAs are often located far away from the transcriptional start site (TSS) and bind tissue-specific TFs; thus, they can regulate differential gene expression [59]. In addition, active enhancers are bound by RNAPII, which may suggest their interaction with the promoter [59]. Intronic lncRNAs originate from intronic regions without overlapping any exons. Sense lncRNAs and antisense lncRNAs may overlap one or more exons of another transcript on the same or opposite strand, respectively; thus, it is able to regulate neighboring a sense gene [67]. Furthermore, bidirectional lncRNAs are transcripts that share the promoter of nearby protein-coding genes on the opposite strand that initiate transcription in close genomic proximity [68].

In addition, lncRNAs are classified based on their regulatory action in (i) cis, which have an impact on the expression and/or chromatin state of surrounding genes; and (ii) trans, which leaves the site of transcription and perform regulatory functions throughout the cell [69].

LncRNAs are expressed in various immune cells, including neutrophils, T lymphocytes, B lymphocytes, macrophages, dendritic cells, and NK cells [68]. Focusing specifically on the skin, lncRNAs emerged as essential regulators of epidermal development, keratinocyte differentiation, melanocyte functions, as well as differentiation and activation of immune cells [68,70]. However, the complexity of multilevel regulation involved in these processes remains unknown [71]. The latest research data have displayed the implication of lncRNAs in various physiological and pathological processes. Nonetheless, the function of the majority of lncRNAs remains unknown [72]. 

Besides lncRNAs, miRNAs, circRNAs, and piRNAs have been tremendously studied and confirmed to modulate gene transcription through pivotal activities in a versatile regulation network [54,55]. miRNAs are epigenetic regulators ~22 nucleotides in length and function as intracellular regulators of gene expression at the post-transcriptional level by inducing transcription degradation or retarding RNA transferase activity through binding to a 30-untranslated region (30-UTR) of target mRNA [12], modification of histone [12], or modulation of methylation in the DNA promoter regions [12,73]. A single miRNA can regulate and/or silence the expression of hundreds of genes, while multiple miRNAs can regulate the expression of a single gene [74,75]. circRNAs are a newly recognized group of ncRNAs characterized by distinctive profiles of conservation, stability, specificity, and complexity [54]. CircRNAs are products of hnRNA back splicing, and the resulting RNAs represent covalently closed circles; thus, they are devoid of terminal RNA cap structures and poly(A) tails [54,76]. However, within this group, few of them have peptide- or protein-coding potential [77]. These circRNAs work as efficient miRNAs sponges in both physiological and pathological processes [54,77]. Moreover, they are able to regulate transcription, splicing, and expression of a parental gene directly by binding to Pol II [54,78]. Additionally, they may interact with proteins [79] or form pseudogenes by retrotranscribing and integrating them into the genome [54,80]. Increased levels of circRNAs in human body fluids can be used as biomarkers of various diseases, including cancer [75,81]. piRNAs are 26–32-nucleotides-long ncRNAs that play a crucial role in development, epigenetic regulation, and transposon silencing [75,82]. piRNAs are regulators of gene expression primarily present in germ cells, and demonstrate a strong tendency for uridine (U) at the 5′ end [75].

### 2.2. Molecular Mechanisms of LncRNAs

To date, several mechanisms of the molecular functioning of lncRNAs are proposed: (i) chromatin modification; (ii) transcription control; and (iii) post-transcriptional regulation [9,83]. LncRNAs are able to regulate chromatin modification, which has an impact on gene expression [9]. The lncRNA that acts as a scaffold is *HOX* antisense intergenic RNA (HOTAIR). It recruits chromatin-modifying complexes, i.e., polycomb repressive complex 2 (PRC2), to the HOXD locus and integrates target genes’ H3K27 methylation and H3K4me2 demethylation. It also contributes to modulating a repressive chromatin state [84], which has been involved in the pathogenesis of certain types of cancer, including breast cancer, lung cancer, and colon cancer [9,85].

Another mechanism of lncRNAs function modulates transcription processes in both lncRNAs transcript sequence-dependent and transcription- or splicing-dependent manners [9]. Therefore, they play a role as transcription factors, coregulators at the promoter regions of specific genes, and transcriptional enhancers [9]. For instance, the lncRNA Blustr regulates the expression of its neighboring protein-coding gene, Sfmbt2, by selecting polymerases and chromatin modifiers in an independent mechanism of the sequence of Blustr itself [86]. Another example is the lncRNA THRIL, which modulates TNF-α expression by interacting with hnRNP L [87]; thus, it affects the ability to bind to the promoter region of target genes. Besides, lncRNA Lethe, which is associated with the NF-kB p65 (RelA) subunit, may inhibit its binding to the promoters of TNF-α, IL-6, and IL-8 [9,88]. In addition, lncRNAs may act as an enhancer element. The gene Cdkn1b is positively regulated through the Cdkn1b (Lockd) locus, which suppresses many enhancer-like elements [89].

Noteworthy, lncRNAs play a significant role in post-transcriptional regulatory networks. Research studies demonstrated that lncRNAs work as competitive endogenous RNAs (ceRNAs), or miRNAs sponges, to decrease the miRNAs levels; thus, they regulate the expression of their target genes [90]. The binding to miR-133 and miR-135 may be a way to control the expression of MAML1 and MEF2C, i.e., genes involved in muscle differentiation [90]. On top of that, lncRNAs are also involved in proteins and influence post-translational modifications such as phosphorylation [91] or ubiquitination [92]. For instance, STAT3 phosphorylation is modulated by lnc-DC [91]. Another example is that the lncRNA NRON controls the dephosphorylation and nuclear import of activated T-cell nuclear factor (NFAT) by producing an NRON–NEAT complex [9,93].

It has been shown that lncRNAs act as molecular sponges of miRNAs by binding to miRNAs via complementary sequences [94,95]. Moreover, miRNAs are a group of well-known small ncRNAs capable of regulating/modulating gene expression by binding to the 3′-untranslated regions (3′-UTR) of target mRNAs [95,96]. In addition, if they compete for shared binding sites, lncRNAs may eliminate the inhibitory effect of miRNAs on gene expression [95]. Thus, the lncRNA-miRNA-mRNA regulatory axis exhibits a crucial way to regulate gene expression [95,96].

## 3. LncRNAs in Systemic Sclerosis

LncRNAs regulate gene expression and may interact with the SSc pathogenesis. Exposure of the skin to several hazardous biochemical substances causes various changes, including the deregulation of ncRNAs expression and functioning [75]. Their role in the context of skin differentiation [97], endothelial damage, inflammation [98], innate and adaptive immunity [99,100], cytotoxicity, and cancers [101] is extensively studied.

**Figure 2:**
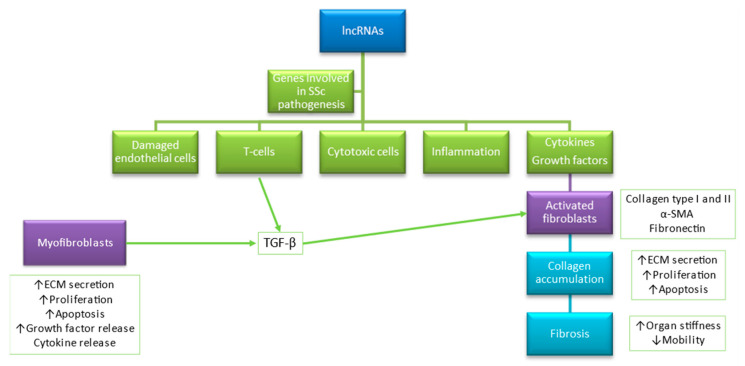
Accumulating evidence shows that lncRNAs play a role in modulation genes related to endothelial function, immunity, inflammation, and cytotoxicity. Based on [15,75].

SSc is an autoimmune connective tissue disease characterized by changes in the immune system, microvasculature, and fibrosis [1]. Noteworthy, 676 deregulated non-coding genes of overall 4901 genes with a fold change of >1.5 and a false discovery rate of <5% were found in SSc patients versus controls by Messemaker et al. [6], and up-regulated genes have a potential role in immunologic, cell adhesion, and keratin-related processes [6]. lncRNAs are associated with vital functions within the skin by regulating the expression of genes involved in splicing [75], modification of chromatin structure [75], protein translation, and post-translational modifications in healthy skin as well as pathological conditions [75,102], particularly in (i) epidermal development; (ii) keratinocyte differentiation; (iii) melanocyte functions; as well as (iv) differentiation and activation of immune cells (Figure 2) [75,102].

For instance, anti-differentiation non-coding RNA (ANCR) and terminal differentiation-induced non-coding RNA (TINCR) were found to be significant in controlling epidermal differentiation [103]. ANCR negatively regulates epidermal differentiation by suppressing premature differentiation in the basal layer of the epidermis [75]; thus, the loss of its progenitor cells induces differentiation [75,102,104]. TINCR is mostly overexpressed in the differentiated epidermal layer and promotes the stability of mRNAs, which is vital for terminal keratinocyte differentiation. The mechanism of action is through a complex production with the RNA-binding protein STAU1. Therefore, its downregulation leads to a sudden loss in the expression of terminal differentiation genes [75,104].

Further, SPRIGHTLY (SPRY4 intronic transcript 1, SPRY4-IT1), a 703 bp cytoplasmic intronic lncRNA, may modify melanocyte functions by stimulating their proliferation while another lncRNA, UCA1, suppresses melanogenesis [75]. Another 743 bp cytoplasmic lncRNA, PlncRNA-1 (also known as CBR3-AS1), is able to target the TGF-β1-Wnt/β-catenin signaling pathway; therefore, it promotes growth, proliferation, and differentiation of human hair follicle stem cells (HFSCs) [75]. In addition, lncRNA H19, RP11-766N7.3, and HOTAIR play an essential role in dermal papilla cells via inhibiting the Wnt/β-catenin signaling pathway [91].

The disruption in the immune system and excessive production of mainly type I collagen (COL1) and several extracellular matrix proteins by dermal skin fibroblasts are crucial in SSc pathogenesis [1]. Recently, Wang et al. found deregulated lncRNA TSIX in SSc dermal fibroblasts both in vivo and in vitro [105]. TSIX siRNA reduced the mRNA expression and impacted the stability of type I collagen in normal and SSc fibroblasts [105,106]. Thus, it has been identified in the regulation of the TGF-β signaling pathway [105,106]. Moreover, it plays a significant role in the constitutive upregulation of collagen in these cells [105]. The expression of the TSIX gene is known to be involved in the X-chromosome inactivation, and it is lost in a variety of human diseases such as female breast or ovarian cancers [107]. Therefore, it may suggest the predominance of autoimmune diseases in the female. Moreover, the deregulated lncRNAs were found in the skin of patients with psoriasis [40].

Another lncRNA involved in regulating TGF-β–driven tissue fibrosis is the newly discovered H19X [108,109]. H19X (also known as MIR503HG) is an intergenic lncRNA found on chromosome X. It is present in several human fibrotic diseases, particularly modulated in dermal fibroblasts, rheumatoid arthritis synovial fibroblasts, and fibroblast-like cells, such as pulmonary artery smooth muscle cells [108]. Research studies revealed the pro-fibrotic effects of H19X by mediating the TGF-β–induced ECM synthesis and differentiation, as well as in ECM-producing myofibroblasts [108,109]. In addition, H19 has a pro-differentiation effect on keratinocyte differentiation [110]. Moreover, Pachera et al. found a DDIT4L as an effector of the H19X-driven impact on collagen production [108].

lncRNAs play a critical role in the healing of wounds in human skin. LOC105372576 (also known as WAKMAR1) is DNA methyltransferase (DNMT) associated with lncRNAs [75], promoting keratinocytes motility and re-epithelialization in wound healing [75]. One experimental study proves that lncRNAs are associated with the impaired wound healing process in diabetes; it has been shown that H19 promoted fibroblast activation and proliferation to attenuate wound healing in mice [111]. In addition, antisense ncRNA in the INK4 locus (ANRIL) has been established to enhance the lymphangiogenesis wound healing process [75], particularly by modulating miR-181a-Prox1 (Prospero homeobox 1) expression [75,112].

Zhao et al. demonstrated that overexpression of lncRNA, HIFα-antisense RNA1 (HIFα-AS1), enhanced the expression of caspase 3, caspase 8, and Bcl-2 in vascular smooth muscle cells (VSMCs) of SSc patients [12,113]. These factors altered proliferation and decreased apoptosis of VSMCs in SSc patients complicated with thoracoabdominal aortic aneurysm [12,113].

Another study carried out in SSc myofibroblasts in vitro, and SSc skin biopsies in vivo displayed the high levels of HOTAIR, a scaffold lncRNA modulating an enhancer of zeste homolog 2 (EZH2) to induce H3K27me3 in specific target genes [114]. Tsou et al. found upregulation of the histone methyltransferase EZH2 and H3K27me3 in ECs from patients with dcSSc ECs compared with healthy controls [115]. EZH2 is the catalytic component of the polycomb repressive complex 2 that represses gene transcription through catalyzing H3K27me3 [115]. Its role is inhibition of dermal ECs angiogenesis [115]. The ability of EZH2 to suppress angiogenesis in ECs of patients with dcSSc was through activating the Notch pathway. Thus, the inhibition of EZH2 led to upregulation of several NOTCH-related genes [115]. Of note, one of the genes, called DLL4, is a NOTCH ligand, and played an essential role in promoting angiogenesis while cells were treated with DZNep [115]. In addition, monocytes treated with the histone methyltransferase inhibitor DZNep and TLR8 agonist ssRNA displayed a pro-fibrotic phenotype along with increased expression of TIMP-1 and Fra2 genes [115]. Transfection of miR-5196 that targets FRA2 in DZNep/ssRNA-treated monocytes significantly decreased expression of FRA2 and TIMP1 [115]. Therefore, it suggests that epigenetic dysregulation in monocytes may play a role in subsequent fibrosis and inflammatory response in patients SSc [116]. Wasson et al. determined that HOTAIR overexpression dependent on EZH2 in dermal fibroblasts increases the expression of collagen and α-SMA [114]. Moreover, they demonstrated a decrease in miRNA-34a expression and, finally, NOTCH pathway activation through the methylation and repression of Notch’s expression negative regulator, miRNA-34a [114].

According to Messemaker et al., three primaries antisense lncRNAs may be involved in SSc pathogenesis, including CTBP1, AGAP2, and OTUD6B [6]. In RNA sequencing (RNA-seq) of skin biopsy samples, they found that 257 antisense lncRNAs are differentially expressed in SSc patients than in healthy subjects [6]. Overexpression of CTBP1 perturbs epidermal and hair follicle homeostasis suggesting its role in skin pathogenesis and wound healing [117]. Furthermore, AGAP2 was found to be involved in cell migration and repress transcription via interaction with EZH2 and lysine-specific histone demethylase 1 (LSD1) in cancer cells [118]. On the other hand, downregulation of OTUD6B is linked to cytokine stimulation and cell proliferation in B cells [119]. Another RNA-seq study presented the substantial deregulation of OTUD6B-AS1 in SSc skin biopsies [120]. After treatment with the platelet-derived growth factor, OTUD6B-AS1 was significantly down-regulated in both fibroblasts from SSc patients and healthy controls [2]. This study also revealed that OTUD6B-AS1 leads to reduced proliferation, suppressed apoptosis, and increased cyclin D1 expression. Therefore, it was found that regulating apoptosis and fibrosis via cyclin D1 expression might be a novel mechanism in the course of SSc [120]. Moreover, OTUD6B-AS1 knockdown exerted a suppressive effect on Wnt/β-catenin signaling via the down-regulation of glycogen synthase kinase-3β interaction protein (GSKIP) [95], suggesting a functional fibrotic effect. Of note, the lncRNA MIR3142HG mediates a pro-fibrotic response to IL-1 in idiopathic pulmonary fibrosis fibroblasts [15,121].

Dolcino et al., in a study carried out in peripheral blood mononuclear cells (PBMCs), isolated from 20 SSc patients and 20 healthy subjects, identified significantly down-regulated lncRNA involved in pathogenetically relevant molecular pathways of SSc [43]. The ncRNA00201 is a heterogeneous nuclear ribonucleoprotein U processed transcript that has been presented as a regulator of tumor proliferation, and its gene target, heterogeneous nuclear ribonucleoproteins C (hnRNPC), is considered as an SSc-associated auto-antigen [43]. Bioinformatic analysis revealed that ncRNA00201 was predicted to modulate 26 miRNAs that affect genes involved in SSc pathogenesis [122].

Moreover, they found that lncRNAs may be involved in the upregulation of genes belonging to the type α- and β-interferon signaling pathways, as well as the increased regulation of interleukin-6 (IL-6), a cytokine playing a major role in inflammatory and autoimmune diseases [43]. In addition, their bioinformatic analysis revealed that ncRNA00201 targets 26 miRNAs, which are regulators of genes such TGF-β receptor, endothelin, epidermal growth factor (EGF) receptor, sphingosine 1 phosphate receptor 1 (S1P1), activin receptor-like kinase 1 (ALK1), ras homolog family member A (RhoA), class I phosphoinositide 3-kinase (PI3K), p38 mitogen-activated protein kinase (MAPK), mammalian target of rapamycin (mTOR), and Toll-like receptors (TLRs) pathways [43]. This finding provides an insight into the SSc pathophysiology.

In the experimental study, Mariotti et al. found NRIR (a negative regulator of the IFN response) lncRNAs associated with the antiviral response, and an interferon synthesis in human monocytes response to TLR4 activation by LPS [123]. A total of 1278 up-regulated and 534 down-regulated lncRNAs were identified by RNA-seq in 46 SSc patients in different stages of their disease compared to 18 healthy subjects [123]. Of note, NRIR was found significantly up-regulated in SSc monocytes in vivo [123]. Besides, it affects the IFN-related chemokines (CXCL10 and CXCL11) associated with SSc pathogenesis [123]. Thus, the dysregulation of NRIR in SSc monocytes might contribute to the aberrant IFN response observed in SSc patients [2,123].

**Figure 3:**
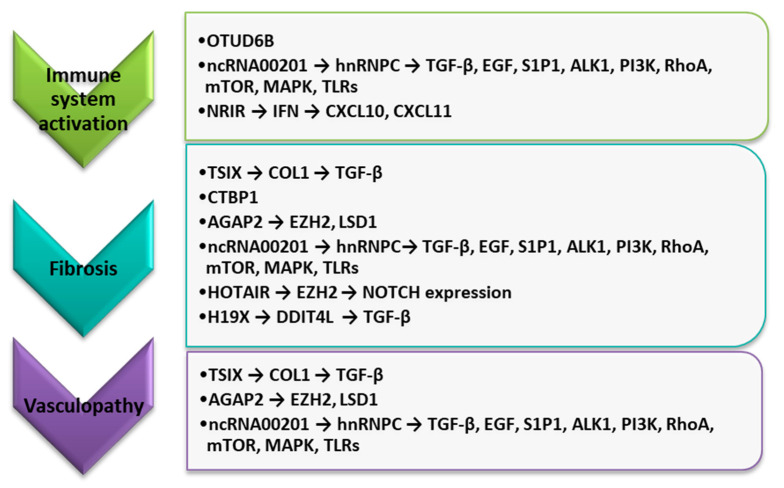
The potential role of selected lncRNAs in the course of SSc pathogenesis. ALK1, activin receptor-like kinase 1; COL1, type I collagen; CXCL10, C-X-C motif chemokine ligand 10; CXCL11, C-X-C motif chemokine ligand 10; EGF, epidermal growth factor; EZH2, enhancer of zeste homolog 2; HOTAIR, *HOX* transcript antisense RNA; IFN, interferon; LSD1, lysine-specific histone demethylase 1A; MAPK, p38 mitogen-activated protein kinase; mTOR, mammalian target of rapamycin; PI3K, class I phosphoinositide 3-kinase; RhoA, Ras homolog family member A; S1P1, sphingosine 1 phosphate receptor 1; TGF-β, tumor necrosis factor-beta; TLRs, Toll-like receptors.

The recent study of Abd-Elmawla et al. demonstrates that lncRNAs in plasma such as ANCR, SPRY4-IT1, HOTTIP, and TINCR are cutting-edge candidate biomarkers for SSc based on their differential expression and correlation with disease clinical manifestations [70]. However, the expression of SPRY4-IT1 was used as a biomarker for the SSc diagnosis and to discriminate its subtypes [70]. Unfortunately, it requires further study in a bigger cohort and external validation. The potential role of lncRNAs in SSc pathogenesis is presented in Figure 3.

However, their role in SSc pathogenesis remains poorly explained. Selected lncRNAs and their pathogenic role in SSc are presented in Table 1 [6].

## 4. LncRNAs as Biomarkers for Diagnosis, Prognosis, and as Targets for Gene Therapy

Because lncRNAs play essential regulatory roles, and their misregulation is associated with several pathologies, the screening of lncRNAs as potential therapeutic agents is being developed. Currently discovered lncRNAs with a potential role in pathogenesis were derived from RNA-seq assemblies [66,127] and the 5′ ends of their transcript models [35,128]. These studies and other functional studies revealed hundreds of potential candidates of epigenetic-modifying drugs in SSc, including DNMT inhibitors (5-azaC and 5-aza), HDAC inhibitors (TSA and MC1568), a HAT inhibitor (SGC-CBP30), SIRT activators (resveratrol, SRT1720, and hexafluoro), EZH2 inhibitors (DZNep and GSK126), a JMJD3 inhibitor (GSKJ4), BET bromodomain inhibitor (JQ1), and miRNAs [115]. Other drugs include ciprofloxacin and retinoic acid (target DNMTs), as well as imatinib and bortezomib (target miRNAs) [16,115].

DNMT inhibitors include azacytidine (5-azaC) and 5-aza-2′-deoxycytidine (5-aza) that incorporate into DNA during replication and sequester DNMTs [115]. Treatment with 5-aza and HDAC inhibitor trichostatin A (TSA) leads to increasing the BMPRII levels in SSc ECs; thus, it could potentially decrease endothelial cell apoptosis [129]. Moreover, it has immune-modulatory effects because 5-azaC is able to stimulate FOXP3 expression and Treg production in CD^4+^ T cells from SSc patients [130]. In dermal fibroblasts, inhibition of DNMTs showed extended anti-fibrotic effects by improving anti-fibrotic activity of transcription factors, i.e., FLI1 and KLF5 [131], and Wnt antagonists, i.e., DKK1 and SFRP1 [132]. On the other hand, suppression of DNMTs prevented bleomycin-induced skin fibrosis in mice [132].

Trichostatin A belongs to HDAC inhibitors and has a beneficial effect as a selective inhibitor for class I and II HDACs in SSc [115]. It prevents the catalytic activity of HDACs by chelating its cofactor zinc ion. TSA administered in bleomycin-treated mice stopped ECM accumulation in the skin [133], as well as efficacy in downregulating fibrotic-related genes, such as COL and Wnt inhibitor WIF1, in dermal fibroblasts isolated from SSc patients [115,133]. Another drug, inhibitor JQ1, blocks histone acetylation via inhibition of histone reader BET bromodomain in dcSSc fibroblasts, efficiently suppressed by the activation of the TGFB2 enhancer, thereby down-regulated TGFB2 and COL1A1 in these cells [115,134]. It also decreased collagen and altered MMP-1 in SSc skin explants [115,134].

In addition, resveratrol and SIRT1720 activate SIRT1, abrogated fibrotic responses in SSc dermal fibroblasts, and bleomycin-induced skin fibrosis in mice [135].

Specific miRNAs, such as let-7a and topical antagomir-155, have been involved in treating SSc fibrosis [136,137]. The intraperitoneal injection of let-7a combined with atelocollagen resulted in the overexpression of let-7a in the skin with a concomitant decrease in collagen production [136]. Moreover, the topical application of miR-155 antagonist decreased the production of collagen in a mouse model [133,135]. The use of bortezomib, a proteasome inhibitor, down-regulates pro-fibrotic miR-21 (up-regulated in fibroblasts of SSc patients) and blocks TGF-β-induced fibrosis in an SSc animal model [138]. Moreover, Regulus therapeutic also has a miRNA-21 antagomir in trials for Alport syndrome; hence, this is another SSc-relevant oligonucleotide therapeutic that should be closely monitored [11]. In a recent study, miRNA-29 mimic (Remlarsen) showed promising anti-fibrotic effects by suppressing collagen expression and development of fibroplasia in incisional skin wounds [139], suggesting its therapeutic option for cutaneous fibrosis [138]. Currently, miRagen therapeutics that have a miR-29 mimic went into clinical trials for SSc with the results eagerly anticipated [15].

Another therapeutic option may be retinoic acid, which is the active metabolite of vitamin A [115]. It can demethylate the promoter region of FOXP3, leading to an increase in FOXP3 expression in SSc CD^4+^ T cells [87].

In addition, they may be potential biomarkers in the diagnosis and prognosis of disorders. It was found that increased plasma levels of TINCR, HOTTIP, and SPRY4-IT1 and decreased levels of ANCR were observed in SSc patients compared to healthy control. Moreover, SPRY4-IT1 and HOTTIP were positively correlated with mRSS. SPRY4-IT1 and ANCR were also associated with PAH. Moreover, SPRY4-IT1, HOTTIP, ANCR, and TINCR in plasma seem to be candidates for biomarkers of SSc. Moreover, SPRY4-IT1 may be helpful to predict the risk of SSc and define subtype [70]. Clinical studies show that miRNAs expression profiles in cell-free serum from patients with different autoimmune diseases, including SSc, systemic lupus erythematosus, rheumatoid arthritis, and mixed connective disease [140]. For instance, Rusek et al. have reported an upregulation of novel serum miRNA-4484 associated with increased MMP-21 expression in SSc [141]. Furthermore, Chouri et al. have reported that miRNA-483-5p in serum is a potential driver of fibrosis in SSc [142]. These findings may suggest that circulatory miRNAs profile is useful as biomarkers and pathogenic indicators for SSc.

Another option is using lncRNAs for gene therapies. However, the whole regulatory network level should be established along with the functions and mechanisms of lncRNAs to be approved in clinical settings [143]. Therefore, the development of this technology requires some extended studies, and more clinical data will advance lncRNAs therapeutic applications [143]. Utilizing ncRNA mimics or inhibitors as tools for targeting the lesional focus will also become a novel therapeutic strategy in the treatment of SSc.

Furthermore, the use of small molecules to target lncRNAs might be a way of treatment, but it requires the identification of relevant RNA motifs with specific structural complexity of lncRNAs to bind lncRNAs with high affinity and specificity [144]. However, this knowledge is still limited [47].

On the other hand, lncRNAs may be molecules of choice for personalized medicine in the future, considering their tissue and disease specificity. The gene expression therapy to increase expression levels or reduce inflammatory response relies on promoter design [145] and the use of the FANTOM5-rich database of promoter usage with tissue specificity, promoter structure, and promoter activities [146]. For instance, 111 lung cancer-associated lncRNAs were identified in RNA-seq analysis of a noninvasive lung cancer cell line (CL1-0) and a more metastatic prone sub-clone (CL1-5). Moreover, the finding confirmed that the lung cancer metastasis-associated lung adenocarcinoma transcript 1 (MALAT1) and the lncRNA smoke- and cancer-associated lncRNA-1 (SCAL1) are good candidates [147]. In addition, it has been demonstrated that the lncRNA-JADE knockdown represses histone H4 acetylation in the DNA damage response pathway; therefore, the breast tumor growth in vivo in mice was reduced [148]. At the time, there is no such study in the autoimmune disease field, particularly in systemic sclerosis.

Over the past few years, another cutting-edge epigenetic engineering called epigenetic editing, by use of CRISPR–Cas9 system, has been proposed as a novel epigenetic editing module for inhibiting aberrant ncRNAs regulation since it is acting as a highly efficient site-specific DNA binding domain [12,149]. The different versions of CRISPR–Cas-engineered molecules allow the deletion, inhibition, or activation of lncRNA-encoding genes, as well as the degradation of the transcripts themselves [47]. However, targeting lncRNAs using CRISPR–Cas is more difficult than targeting protein-coding genes; therefore, the therapeutic application of CRISPR–Cas systems at lncRNA loci is still behind in the personalized medicine.

## 5. Conclusions and Future Perspectives

Transcriptomic studies have shown that most of the human genome is expressed in cell-specific patterns and produces several non-coding RNAs. Recent investigations have revealed that intracellular and extracellular ncRNAs, including lncRNAs, miRNAs, and circRNAs, are the key molecules for post-transcriptional regulation of mRNA expression. LncRNAs are important players in many physiological and pathological processes through modulating the transcriptional output, mRNA stability, and protein functions in the cell. Since the lncRNAs research in the autoimmune diseases field is developing, future research should focus on finding the DNA, RNA, and protein targets for lncRNAs that may contribute to SSc pathogenesis. It is observed that these molecules are correlated to clinical manifestation and disease activity, suggesting that their profiling could reveal its function as potential diagnostic and/or prognostic biomarkers as well as the targets of new strategies for the treatment of SSc. However, only a few of them were found. It required further verification, confirming the function in the process and precisely explain the molecular pathogenesis. Technological advances, including new models, single-cell technologies, and gene editing, could provide new insights into the pathogenesis of fibrotic diseases, including SSc, identification of epigenetic biomarkers, and the development of novel epigenetic drugs for their treatment; therefore, the initiation of clinical trials for SSc patients may start shortly.

## Figures and Tables

**Table 1:** Selected lncRNAs and their pathogenic role in SSc [6].

LncRNA	Full Name	Regulation	Function	Ref.
AGAP2	ADP-ribosylation factor GTPase activating protein 2	Up	Involved in focal adhesion and cell migration	[6,124,125]
CTBP1	C-terminal-binding protein 1	Up	A C terminal binding protein acting as a transcriptional corepressor Plays a role in epidermal development Disrupts skin homeostasis	[6,117,126]
H19X	MIR503HG, intergenic lncRNA		TGF-β–induced ECM synthesis as well as differentiation and survival of ECM-producing myofibroblasts Regulates DDIT4L gene expression Role in dermal papilla cells via suppressing the Wnt/β-catenin signaling pathway	[91,108,109]
HIFα-AS1	HIFα-antisense RNA1	Up	Enhanced the expression of caspase 3, caspase 8, and Bcl-2 in vascular smooth muscles cells	[12]
HOTAIR	*HOX* transcript antisense RNA	Up	Pro-fibrotic activation and myofibroblast transformation of dermal fibroblasts in vitro Induces EZH2-dependent increase in collagen and α-SMA expression in vitro Inhibition of miRNA-34a expression NOTCH pathway activation Role in dermal cells via suppressing the Wnt/β-catenin signaling pathway	[91,114]
ncRNA00201 (HNRPU)	Heterogeneous nuclear ribonucleoprotein U	Down	Regulates genes involved in vasculopathy, fibrosis, and autoimmunity	[43]
NRIR	Negative regulator of the IFN response	Up	Regulates IFN response	[123]
OTUD6B	Ovarian tumor domain-containing 6B	Down	A deubiquitinating enzyme; linked to cell proliferation in B cells following prolonged cytokine stimulation	[119]
PlncRNA-1 (also known as CBR3-AS1)	CBR3 Antisense RNA 1		Promotes growth, proliferation, and differentiation of human hair follicle stem cells (HFSCs)	[90]
RP11-766N7.3	-		Role in dermal cells via suppressing the Wnt/β-catenin signaling pathway	[91]
SPRIGHTLY	SPRY4 intronic transcript 1, SPRY4-IT1		Regulation of melanocyte functions by stimulating their proliferation	[88,89]
TSIX	X-inactive specific transcript antisense	Up	Increases stability of type I collagen mRNA	[105]
UCA1	Urothelial Cancer-Associated 1		Suppression of melanogenesis	[88,89]
